# Artificial intelligence optimizes immune rejection prediction and management in heart transplantation: a structured narrative review

**DOI:** 10.3389/fcvm.2026.1790244

**Published:** 2026-05-14

**Authors:** Kaixin Chen, Junlin Lai, Yijie Luo, Chenghao Li, Guohua Wang

**Affiliations:** 1 Department of Cardiovascular Surgery, Union Hospital, Tongji Medical College, Huazhong University of Science and Technology, Wuhan, China; 2 Department of Cardiovascular Surgery, Zhongnan Hospital, Wuhan University, Wuhan, Hubei, China

**Keywords:** artificial intelligence, deep learning, donor-recipient matching, heart transplantation, immune rejection, machine learning, non-invasive monitoring, pathological diagnosis

## Abstract

**Background:**

Heart transplantation remains the definitive therapy for end-stage heart failure, yet long-term outcomes are limited by three core clinical bottlenecks in immune rejection management: imprecise preoperative donor-recipient matching, overreliance on invasive endomyocardial biopsy (EMB) for postoperative rejection surveillance, and high inter-observer variability in manual pathological diagnosis of rejection. Artificial intelligence (AI) has emerged as a promising tool to address these gaps, but the methodological quality and clinical translation readiness of supporting evidence have not been comprehensively synthesized.

**Methods:**

This structured narrative review synthesized original research published between October 1, 2020, and October 1, 2025, identified via a targeted PubMed search and manual reference screening. Two independent reviewers performed study selection and data extraction, with discrepancies resolved by consensus. Common methodological limitations across included studies were synthesized qualitatively.

**Results:**

A total of 42 studies were included in the final qualitative synthesis. Preoperatively, 3D-Convolutional Neural Networks (3D-CNNs) enabled automated, accurate total cardiac volume (TCV) measurement for anatomical matching, while machine learning models identified non-linear synergistic risk factors for postoperative adverse events, outperforming traditional regression models. Postoperatively, AI models integrating non-invasive biomarkers (gene expression profiles, extracellular vesicles, donor-derived cell-free DNA) showed high diagnostic accuracy for rejection, with one single-center retrospective study estimating a 56.8% reduction in unnecessary EMB procedures (prospective clinical validation is still required). For pathological diagnosis, AI models improved the sensitivity of high-grade acute cellular rejection (ACR) detection from 39.5% to 74.4% compared with manual assessment, generative adversarial networks (GANs) addressed rare rejection sample scarcity with a rejection region detection AUROC of 98.84%, and explainable AI tools aligned model decisions with pathologists' judgment. The overall methodological quality of included studies was suboptimal, with most studies limited by single-center retrospective design, small sample size, and lack of independent external validation.

**Conclusions:**

AI has demonstrated promising potential to optimize donor-recipient matching, enable non-invasive rejection surveillance, and standardize pathological diagnosis in heart transplantation. However, most current evidence comes from exploratory, single-center retrospective studies with important methodological limitations that restrict their immediate clinical translation. Future research should prioritize prospective, multi-center clinical validation, standardized biomarker and model reporting, and federated learning data ecosystems to translate AI innovations into routine clinical practice.

## 1 Introduction

Heart transplantation remains the definitive therapy for patients with end-stage heart failure (17). However, the trajectory from preoperative donor-recipient matching to the long-term management of postoperative rejection is fraught with challenges that critically impact transplant success and patient quality of life (18). Preoperatively, traditional matching relies on limited clinical metrics, often failing to capture complex physiological compatibility, leading to suboptimal donor heart utilization and increased complication risks (19). Postoperatively, immune rejection monitoring depends heavily on invasive endomyocardial biopsy (EMB), a procedure associated with procedural risks, diagnostic inconsistency, and limited sensitivity for early detection (20).

Recently, Artificial Intelligence (AI) has emerged as a transformative force in addressing these persistent clinical gaps. Leveraging advanced capabilities in pattern recognition and deep learning, AI can extract latent insights from vast datasets—spanning imaging, pathology, and genomics—facilitating more precise, personalized clinical decision-making (21).

**Figure 1:**
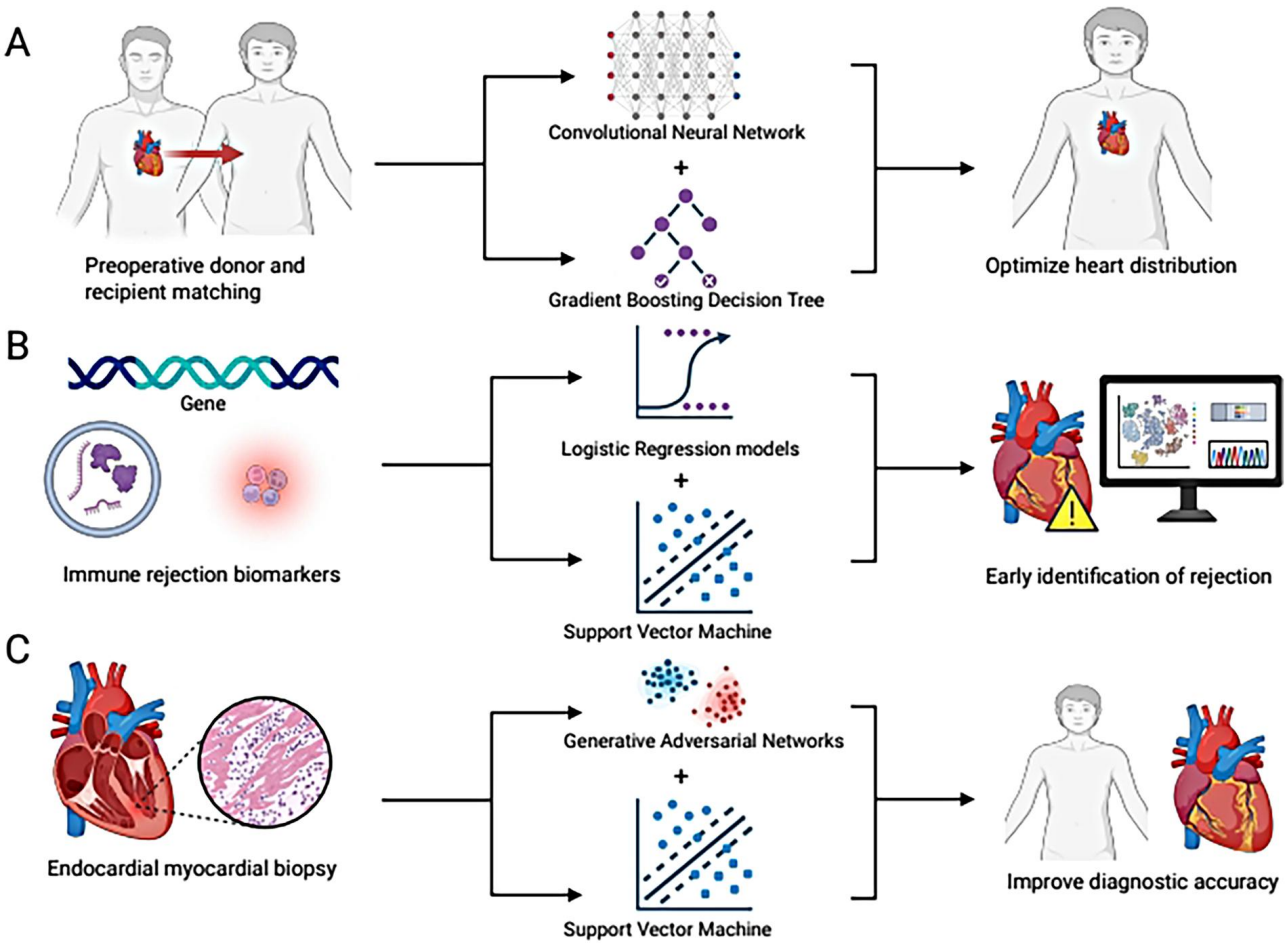
Conceptual framework of artificial intelligence applications in heart transplantation. **(A)** Preoperative donor-recipient matching: 3D-Convolutional Neural Networks (CNNs) quantify total cardiac volume for precise size matching, while gradient boosting decision trees analyze risk factors to optimize allocation and improve donor heart utilization. **(B)** Postoperative rejection warning: Machine learning models (e.g., logistic regression, SVM) integrate biomarkers such as gene expression profiles, extracellular vesicles (EVs), and donor-derived cell-free DNA (dd-cfDNA) to identify rejection early, reducing reliance on invasive biopsies. **(C)** Pathological biopsy image analysis: Generative Adversarial Networks (GANs) and SVMs quantify histological features to improve Acute Cellular Rejection (ACR) grading and Cardiac Allograft Vasculopathy (CAV) prediction, reducing inter-observer variability.

This Structured Narrative Review synthesizes recent advances (2020–2025) in AI applications within heart transplantation. Specifically, we examine three critical domains: (1) the optimization of preoperative donor-recipient matching through precise volumetric measurement and multidimensional risk modeling; (2) the development of non-invasive postoperative rejection screening using novel biomarkers (e.g., gene expression, extracellular vesicles, donor-derived cell-free DNA); and (3) the enhancement of pathological diagnosis via automated image analysis. By critically evaluating these studies, this review aims to elucidate how AI is reshaping the paradigm of rejection prediction and management, while identifying current limitations and future directions for clinical integration. To visualize the scope of this Structured Narrative Review, we present a conceptual framework illustrating AI applications across the three key domains of heart transplantation (Figure 1).

## 2 Materials and methods

This structured narrative review was conducted to systematically synthesize the current state of artificial intelligence applications in immune rejection prediction and management across the full heart transplantation care continuum.

### 2.1 Literature search strategy

A targeted literature search was performed in the PubMed database on October 5, 2025, to identify relevant studies published between October 1, 2020, and October 1, 2025. No language restrictions were applied. The core PubMed search syntax was as follows: (“Heart Transplantation"[MeSH Terms] OR “heart transplant*"[Title/Abstract] OR “cardiac transplant*"[Title/Abstract]) AND (“Artificial Intelligence"[MeSH Terms] OR “AI"[Title/Abstract] OR “machine learning"[Title/Abstract] OR “deep learning"[Title/Abstract] OR “neural network*"[Title/Abstract] OR “convolutional neural network*"[Title/Abstract]) AND (“immune rejection"[Title/Abstract] OR “allograft rejection"[MeSH Terms] OR “graft rejection"[Title/Abstract]) AND (“2020/10/01"[Date—Publication]: “2025/10/01"[Date—Publication]).

In addition, we manually screened the reference lists of all eligible studies and relevant review articles to identify additional records not captured by the database search.

### 2.2 Study selection and eligibility criteria

The initial search yielded 169 studies. All records were reviewed in two stages: initial screening of titles and abstracts, followed by full-text review.

The inclusion criteria were: (1) original research articles published within the specified five-year period; (2) studies involving human subjects; and (3) content directly evaluating AI or machine learning models for predicting cardiac transplant immune rejection (including donor-recipient matching, non-invasive rejection warning, and pathological image analysis).

The exclusion criteria were: (1) studies not directly related to heart transplantation; (2) research involving other cardiac replacement therapies (e.g., ventricular assist devices); (3) non-human studies (animal or *in vitro* experiments); content directly evaluating AI or machine learning models for predicting cardiac transplant immune rejection (including donor-recipient matching, non-invasive rejection warning, and pathological image analysis) and cardiac allograft vasculopathy (CAV), the main form of chronic rejection. (4) conference abstracts, editorials, and reviews; and (5) non-English literature. Ultimately, 42 articles meeting these criteria were included in the final synthesis.

### 2.3 Data extraction and synthesis

A standardized data extraction form was developed *a priori*, and data extraction was independently performed in duplicate by two reviewers, with all discrepancies resolved via consensus discussion or arbitration by a third senior investigator. The following variables were extracted from each included study: basic study characteristics, cohort features, AI model details, core performance metrics, clinical outcomes, and study limitations.

We synthesized the included studies into three clinically relevant domains: preoperative donor-recipient matching, postoperative non-invasive rejection surveillance, and automated pathological biopsy image analysis, to provide a structured overview of current advances and limitations.

### 2.4 Institutional review board statement

This review is based exclusively on previously published peer-reviewed literature, with no direct use of human patient data. Therefore, ethical review and approval were waived by the Institutional Review Board of Union Hospital, Tongji Medical College, Huazhong University of Science and Technology.

### 2.5 Methodological limitation synthesis

We systematically extracted and synthesized common methodological limitations across all included studies, focusing on factors that impact the generalizability and clinical translation of AI models. Key limitations evaluated included study design (prospective vs. retrospective), sample size, validation strategy (internal vs. external), and reporting transparency. No formal per-study risk-of-bias grading was performed, as this is not required for narrative reviews.

### 2.6 Sex and gender analysis

In compliance with the Sex and Gender Equity in Research (SAGER) guidelines, we assessed the reporting of sex and gender in the primary studies. Data regarding participant sex and gender distributions were extracted from the included studies where available to evaluate the representativeness of the training and validation cohorts.

### 2.7 Patient and public involvement (PPI)

This Structured Narrative Review synthesizes previously published literature and did not involve direct patient or public participation in study design, data collection, or analysis. The research agenda was informed by clinical unmet needs in heart transplant rejection management, with the aim of providing evidence to improve patient care and outcomes.

## 3 Relevant sections

### 3.1 Spectrum of AI algorithms employed

Our analysis identified a diverse array of AI architectures applied to heart transplantation. Convolutional Neural Networks (CNNs), particularly 3D-Convolutional Neural Networks (e.g., DenseNet, ResNet), were the predominant choice for processing medical imaging data, utilized extensively for quantifying morphological metrics like Total Cardiac Volume (TCV) and classifying pathological biopsy images (22–25). For structured clinical data, Gradient Boosting Decision Tree (GBDT) algorithms, including XGBoost, CatBoost, and LightGBM, demonstrated superior performance in handling tabular data for risk prediction and donor allocation, outperforming traditional logistic regression in identifying non-linear risk factors (26, 27). Traditional Machine Learning models (Random Forest, SVM, LASSO) remained robust tools for high-dimensional biomarker screening and feature selection (28–33). Notably, recent studies have increasingly integrated Explainable AI (XAI) techniques (SHAP, LIME, Grad-CAM) to demystify the “black box” nature of models, enhancing clinical trust by visualizing decision-making logic (34–39). Furthermore, Generative Adversarial Networks (GANs) have emerged as a novel solution for data augmentation, effectively addressing the scarcity of rare rejection samples (40, 41). A comprehensive overview of these algorithms and their specific applications is presented in Table 1.

**Table 1:** Overview of AI algorithms and their applications in heart transplant rejection prediction.

Algorithm category	Key architectures/models	Key technical features	Specific applications in heart transplantation
Convolutional Neural Networks (CNNs)	3D-CNN (DenseNet, ResNet), ResNet50, EchoNet-Dynamic	Deep feature extraction from images Automated segmentation Spatial attention mechanisms	Quantifying Total Cardiac Volume (TCV) for size matching Automated grading of pathological biopsies (ACR/AMR) Estimating ejection fraction from echocardiograms
Gradient Boosting Decision Trees (GBDT)	XGBoost, CatBoost, LightGBM	Handling structured/tabular clinical data Feature importance ranking Robustness to missing data	Predicting Primary Graft Dysfunction (PGD) risk Identifying hidden biases in donor heart allocation Long-term rejection risk stratification
Traditional Machine Learning	Random Forest, SVM, Logistic Regression, LASSO	High interpretability Effective feature selection Suitable for smaller datasets	Screening genomic biomarkers (e.g., PBMCs) Classifying rejection based on EV proteins Developing clinical risk scoring models
Explainable AI (XAI)	SHAP, LIME, Grad-CAM, Integrated Gradients	Quantifying feature contribution Visualizing decision logic (heatmaps) “Black box” demystification	Enhancing clinician trust in risk predictions Explaining individual patient risk factors Justifying pathological grading decisions
Generative AI	GANs (Progressive, Heuristic)	Synthetic data generation Latent space analysis Data augmentation	Generating synthetic samples for rare rejection types Overcoming data scarcity in pediatric cohorts Enhancing model training robustness
Auxiliary Technologies	Federated Learning, Transfer Learning, Multiple Instance Learning	Privacy-preserving data integration Cross-organ knowledge transfer Weakly supervised learning	Constructing multi-center data ecosystems Pan-organ rejection modeling (e.g., Kidney-Heart) Utilizing unannotated whole-slide images

### 3.2 Optimization of preoperative donor-recipient matching

AI has significantly advanced the precision of preoperative matching beyond traditional weight-based and single-indicator methods.

Anatomical Matching: Szugye et al. deployed a 3D-Convolutional Neural Networks model to automate Total Cardiac Volume measurement from CT scans. This approach reduced measurement error to 4.5%±3.9% in normal hearts and significantly streamlined the workflow compared to manual segmentation, providing a standardized metric for pediatric size matching (1).

Risk Stratification: Machine learning models have revealed complex, non-linear interactions among donor and recipient variables. Ding et al. demonstrated that a Multilayer Perceptron (MLP) model (AUROC 0.868) outperformed logistic regression in predicting Primary Graft Dysfunction (PGD) by identifying synergistic risk factors, such as the compounded risk of older donor age combined with recipient cardiovascular surgical history (2).

Allocation Efficiency: In donor allocation, Haregu et al. utilized a CatBoost model to quantify “hidden” decision biases, finding that the number of prior rejections of a donor heart was a stronger predictor of acceptance than clinical quality alone. This quantification offers a data-driven basis for optimizing regional sharing protocols (3).

Key findings and clinical implications for preoperative matching are summarized in Table 2.

**Table 2:** Key studies on AI in preoperative donor-recipient matching and allocation.

Study	AI model	Data source/cohort	Key findings	Clinical impact
Szugye et al. (1)	3D-CNN (DenseNet + ResNet)	Pediatric CT scans (*n* = 314)	TCV measurement error: 4.5% (normal hearts) Dice similarity coefficient: 0.94 ± 0.03	Replaces manual segmentation (time reduced from 30 min to <1 min) Provides objective metric for size matching
Ding et al. (2)	Multilayer Perceptron (MLP)	UNOS Database (*n* = 8,008)	PGD prediction AUROC: 0.868 Identified synergistic risks (e.g., donor age >40 + recipient CV surgery)	Reveals non-linear risk superposition effects Superior to logistic regression for PGD risk stratification
Haregu et al. (3)	CatBoost	UNOS Pediatric Data	“Allocation sequence number” identified as key predictor of acceptance Acceptance probability ↓ 47% after >5 rejections	Quantifies “group decision bias” in allocation Supports optimization of regional sharing strategies to reduce waste
Zhou et al. (4)	Random Forest + SHAP	Single-center China (*n* = 381)	AUROC: 0.801 for 1-year mortality Key features: Albumin, Recipient Age, LA Diameter	SHAP force plots provide interpretable, individualized risk explanations Guides preoperative nutritional interventions
Lang et al. (5)	Deep Learning (CNN)	Preoperative Chest CT (*n* = 164)	Sarcopenia at T11 associated with graft loss (HR = 3.86) Rejection risk: 32% (sarcopenia) vs. 20% (control)	Automates frailty assessment from routine imaging Identifies high-risk recipients requiring pre-transplant rehabilitation

### 3.3 Postoperative non-invasive rejection surveillance

AI integration has facilitated the discovery and validation of non-invasive biomarkers, offering alternatives to invasive Endomyocardial Biopsy (EMB).

Genomic and Transcriptomic Markers: Machine learning workflows (e.g., LASSO, Random Forest) have successfully isolated rejection-specific gene signatures (e.g., ALAS2, HBD, KLRD1) from peripheral blood, achieving high diagnostic accuracy (AUC > 0.90) and enabling detection weeks prior to histological manifestation (6, 42, 43). Cross-organ transfer learning further validated robust markers like CXCL9 across kidney and heart transplantation (44).

Extracellular Vesicles (EVs) and donor-derived cell-free DNA: Castellani et al. (7) developed a two-layer Random Forest model based on EV surface proteins in a single-center retrospective cohort (*n* = 100), which achieved 86.5% validation accuracy for differentiating rejection vs. non-rejection, and 95% accuracy for distinguishing ACR from AMR. In this exploratory single-center retrospective study with a small sample size, the model was estimated to reduce unnecessary EMB procedures by 56.8% in low-risk patients. However, this finding has not been validated in prospective multi-center clinical trials, and cannot be extrapolated to routine clinical practice at present (7). Similarly, AI-enhanced analysis of peripheral blood donor-derived cell-free DNA (dd-cfDNA), a non-invasive biomarker, has shown high accuracy in distinguishing rejection subtypes and correlating with graft injury severity. Beyond circulating biomarkers, AI has also advanced molecular analysis of endomyocardial biopsy tissue: the AI-augmented Molecular Microscope (MMDx) gene expression profiling system further improves the accuracy of rejection subtyping and graft fibrosis risk stratification, effectively distinguishing TCMR, AMR, and mixed rejection, and correlating with long-term graft survival (45, 46).

Imaging and Electrophysiology: Deep learning analysis of standard 12-lead ECGs demonstrated high sensitivity (95%) for detecting moderate-to-severe Acute Cellular Rejection, enabling scalable remote monitoring (8). Performance metrics of these biomarker-based AI models are detailed in Table 3.

**Table 3:** Performance of AI models for postoperative non-invasive rejection monitoring.

Biomarker type	Study	AI architecture	Performance metrics	Clinical utility
Gene Expression	Wang et al. (6)	LASSO + Random Forest	AUROC: 0.944 (4-gene signature: ALAS2, HBD, etc.) High calibration accuracy	Enables early warning 2–4 weeks prior to histological signs Reduces missed diagnosis rates
Extracellular Vesicles (EVs)	Castellani et al. (7)	Two-layer Random Forest	Validation Cohort Accuracy: 86.5% (Rejection vs. Non-rejection) Typing Accuracy: 95% (ACR vs. AMR)	Potential to reduce EMB demand by 56.8% Differentiates rejection subtypes non-invasively
ECG Analysis	Adedinsewo et al. (8)	Deep Learning (CNN)	AUROC: 0.84 (Retrospective) Sensitivity: 95% NPV: 99.7% for moderate-severe ACR	Enables scalable remote/home monitoring Detects subtle signal changes invisible to human eye
Urinary Proteomics	Wei et al. (9)	XGBoost	AUROC: 0.71 for CAV diagnosis NRI: 80.5%; IDI: 9.9% vs. clinical models	Significant improvement over traditional CAV risk scores Highlights collagen metabolism pathways
Inflammatory Markers	Feng et al. (10)	Elastic Net Cox Regression	C-index improvement: +0.012 NRI: 17.3% for prognostic prediction	Supplementary value to standard clinical models Cost-effective risk stratification

The Wei et al. study focuses on Cardiac Allograft Vasculopathy (CAV), the leading cause of late graft loss after heart transplantation. It is included here to demonstrate the broader utility of AI in post-transplant complication surveillance, which is an integral component of long-term rejection management.

**Table 4:** Diagnostic performance of AI in pathological biopsy image analysis.

Study	AI model/tool	Diagnostic task	AI performance	Advantage vs. traditional manual assessment
Peyster et al. (11)	SVM (CACHE-Grader)	ACR Grading (ISHLT criteria)	Sensitivity (Grade 2R/3R): 74.4% Concordance with clinical grades: 65.9%	Significantly higher sensitivity than manual assessment (∼39.5%) Consistent across multi-center datasets
Glass et al. (12)	ResNet50 (CNN)	Differential Diagnosis (AMR vs. ACR vs. Healing)	Overall Accuracy: 98% AMR vs. Normal: 99.2% AMR vs. Healing Injury: 99.5%	Overcomes low inter-observer agreement (Kappa 0.10–0.65) in complex mixed rejection cases
Giuste et al. (13)	Progressive GANs + CNN	Pediatric Rejection Detection (Rare samples)	Rejection Region AUROC: 98.84% Biopsy-level AUROC: 95.56%	Solves the “small sample size” bottleneck in pediatric cohorts using synthetic data generation
Lipkova et al. (14)	CRANE (CNN)	Detection, Typing, and Grading	Stable performance across international cohorts Generated attention heatmaps	Provides a comprehensive, explainable diagnosis pipeline Visualizes specific regions of interest for pathologists
Kim et al. (15)	XGBoost (Integrated Rejection Risk Index)	90-day Rejection Risk Prediction	High-risk group event rate: 75.6% Low-risk group event rate: 10.7%	Shifts paradigm from “current diagnosis” to “risk prediction” Integrates histological features with clinical metadata
Seraphin et al. (16)	Multiple Instance Learning (MIL)	Binary Rejection Classification	Cross-validation AUROC: 0.849 External Validation AUROC: 0.716–0.734	Utilizes self-supervised learning on unannotated slides Reduces the burden of pixel-level manual annotation

### 3.4 Automated pathological biopsy image analysis

AI algorithms have demonstrated “super-assistant” capabilities in addressing the subjectivity and low consistency of manual pathological grading.

Diagnostic Consistency and Sensitivity: The “CACHE-Grader” system (SVM-based) improved the sensitivity for detecting high-grade rejection (2R/3R) to 74.4%, significantly surpassing the ∼39.5% sensitivity of manual assessment (11).

Complex and Rare Cases: For differentiating complex pathologies, a ResNet50 model achieved >99% accuracy in distinguishing AMR from Acute Cellular Rejection and healing injury, resolving inter-observer variability issues (12). To address data scarcity in pediatric rejection, Giuste et al. successfully employed Progressive GANs to generate synthetic training data, achieving an AUROC of 98.84% in rejection region detection (13). Similarly, computational analysis of routine biopsies integrating histopathological features has been shown to improve the prediction of Cardiac Allograft Vasculopathy (CAV) (47). The diagnostic performance of AI in pathological biopsy image analysis is evaluated in Table 4.

### 3.5 Common methodological limitations

Most included studies shared similar methodological limitations that restrict their immediate clinical translation.

Among the 26 clinical prediction model studies, only 5 (19.2%) used a prospective design, 7 (26.9%) employed consecutive patient recruitment, 5 (19.2%) appropriately handled missing data, and 9 (34.6%) had a sufficient events per predictor ratio (>10). While 19 studies (73.1%) performed internal validation, only 7 (26.9%) conducted independent external validation.

Among the 16 diagnostic accuracy studies, only 4 (25.0%) were prospective, 5 (31.3%) used consecutive recruitment, 5 (31.3%) implemented blinding of AI model assessors, and 3 (18.8%) blinded reference standard assessors. Twelve studies (75.0%) performed internal validation, but only 4 (25.0%) had independent external validation.

Notably, 92.3% of prediction model studies and 87.5% of diagnostic accuracy studies had low applicability concerns, with study populations and research questions fully aligned with the scope of this review.

## 4 Discussion

### 4.1 Principal findings and clinical implications

This Structured Narrative Review synthesizes the rapidly evolving landscape of AI in heart transplantation, confirming its capacity to address critical bottlenecks across the continuum of care. Our analysis indicates that AI is not merely an incremental improvement but a paradigm shift from experience-based subjective judgment to data-driven precision medicine.

In the preoperative phase, the superiority of AI lies in its ability to handle high-dimensional complexity. Traditional donor-recipient matching, heavily reliant on single metrics like weight or blood type, fails to account for the non-linear interactions between donor and recipient physiological profiles. Our review highlights that AI models, such as 3D-Convolutional Neural Networks for volumetric analysis and machine learning for risk stratification, provide a more granular assessment of compatibility. By identifying synergistic risk factors (e.g., specific donor age-recipient history interactions) and quantifying hidden allocation biases, AI offers a concrete pathway to improve donor heart utilization and reduce primary graft dysfunction.

Postoperatively, the integration of AI with novel biomarkers represents a crucial step toward non-invasive surveillance. The dependency on Endomyocardial Biopsy (EMB) has long been a clinical dilemma due to its invasiveness and sampling errors. The studies reviewed demonstrate that AI classifiers utilizing gene expression, extracellular vesicles, and donor-derived cell-free DNA can effectively filter low-risk patients, potentially reducing the frequency of invasive procedures by over 50%. More importantly, AI's ability to distinguish complex rejection phenotypes (e.g., mixed rejection or Antibody-Mediated Rejection) surpasses the resolution of traditional singular biomarkers.

In pathological diagnosis, AI serves as an objective standardizing tool. The historical challenge of high inter-observer variability in endomyocardial biopsy grading is effectively mitigated by deep learning systems. By automating the quantification of histological features—such as lymphocyte density and stromal architecture—AI ensures diagnostic consistency across centers and provides “super-human” sensitivity in detecting subtle or rare rejection patterns that may be overlooked by visual inspection alone.

### 4.2 Limitations and challenges

Despite these promising advances, several impediments restrict the immediate clinical translation of AI. First, data heterogeneity and scarcity remain significant hurdles. Most models are trained on single-center, retrospective cohorts with limited sample sizes, particularly for rare complications. This raises concerns about algorithmic bias and generalizability to diverse populations. Second, the lack of standardization in biomarker quantification (e.g., EV isolation protocols) introduces noise that hampers model reproducibility. Third, the “black box” phenomenon persists as a barrier to adoption. While Explainable AI (XAI) techniques like SHAP and Grad-CAM are increasingly employed, linking algorithmic “attention” to biological pathophysiology remains necessary to build clinician trust. Finally, ethical and regulatory frameworks for AI diagnostic tools are still nascent, particularly regarding liability in AI-assisted decision-making and data privacy in cross-institutional sharing. Additionally, this review is limited to English-language studies indexed in PubMed, which may introduce publication bias and miss relevant gray literature or studies published in other languages.

### 4.3 Future directions and conclusion

The future of AI in heart transplantation lies in the development of multicenter, federated learning ecosystems. Such platforms would enable the training of robust, generalizable models on global datasets without compromising patient privacy. Furthermore, the next generation of models must move towards multimodality, integrating genomics, imaging, and clinical data into unified predictive engines that mirror the holistic nature of clinical practice.

In conclusion, Artificial Intelligence is reshaping heart transplantation by enhancing the precision of donor matching, enabling non-invasive rejection monitoring, and standardizing pathological diagnosis. While challenges in data standardization and interpretability remain, the transition from “auxiliary tool” to “intelligent partner” is underway. Continued interdisciplinary collaboration is essential to translate these algorithmic innovations into tangible survival benefits for heart failure patients.
